# Outdoor air pollution and emergency department visits for asthma among children and adults: A case-crossover study in northern Alberta, Canada

**DOI:** 10.1186/1476-069X-6-40

**Published:** 2007-12-24

**Authors:** Paul J Villeneuve, Li Chen, Brian H Rowe, Frances Coates

**Affiliations:** 1 Biostatistics and Epidemiology Division, Health Canada, Ottawa, Ontario, Canada; 2 University of Alberta Hospital, 8440-112th Street, Edmonton, Alberta, Canada; 3 Aerobiology Research Laboratories, Ottawa, Ontario, Canada

## Abstract

**Background:**

Recent studies have observed positive associations between outdoor air pollution and emergency department (ED) visits for asthma. However, few have examined the possible confounding influence of aeroallergens, or reported findings among very young children.

**Methods:**

A time stratified case-crossover design was used to examine 57,912 ED asthma visits among individuals two years of age and older in the census metropolitan area of Edmonton, Canada between April 1, 1992 and March 31, 2002. Daily air pollution levels for the entire region were estimated from three fixed-site monitoring stations. Similarly, daily   levels of aeroallergens were estimated using rotational impaction sampling methods for the period between 1996 and 2002. Odds ratios and their corresponding 95% confidence intervals were estimated using conditional logistic regression with adjustment for temperature, relative humidity and seasonal epidemics of viral related respiratory disease.

**Results:**

Positive associations for asthma visits with outdoor air pollution levels were observed between April and September, but were absent during the remainder of the year. Effects were strongest among young children. Namely, an increase in the interquartile range of the 5-day average for NO_2 _and CO levels between April and September was associated with a 50% and 48% increase, respectively, in the number of ED visits among children 2 – 4 years of age (p < 0.05). Strong associations were also observed with these pollutants among those 75 years of age and older. Ozone and particulate matter were also associated with asthma visits. Air pollution risk estimates were largely unchanged after adjustment for aeroallergen levels.

**Conclusion:**

Our findings, taken together, suggest that exposure to ambient levels of air pollution is an important determinant of ED visits for asthma, particularly among young children and the elderly.

## Background

Asthma is a common, heterogeneous chronic lung disease caused by a combination of genetic and environmental influences. It is well recognized that exposure to outdoor air pollution adversely affects respiratory health, even in non-asthmatics [1]. Numerous epidemiologic studies have documented that outdoor air pollution is associated with decreased lung function [2,3], and an increased number of hospital admissions for asthma [4-8]. A series of studies have also reported associations between outdoor air pollution levels and emergency department visits for asthma [9-11], a surrogate measure for asthma attacks.

While children and the elderly have been identified as population subgroups particularly sensitive to the harmful effects of air pollution [12,13], risk estimates for asthma obtained from hospital-based studies have not been consistent. These differences may be attributed, in part, to variations in the pollution mix between the urban centers that were examined, or other uncontrolled factors (e.g., pollen) that vary seasonally. In addition, studies that have investigated associations in children have, for the most part, used broadly defined age-groups. Differences between infants and adolescents with respect to activity patterns, lung development, and immune systems suggest that associations between outdoor air pollution and respiratory health could vary in important ways [12].

In this study, we use a time-stratified case-crossover study design to examine the effects of outdoor air pollution on the daily number of ED visits in the census metropolitan area of Edmonton, Alberta. This area, that has a population of nearly one million persons, has ambient pollution levels that are influenced by several factors. These factors include emissions from coal fired power plants that are approximately 65 kilometers to the west, urban vehicular traffic, and petrochemical refineries and a variety of other industries to the east. The primary objective of this investigation was to examine variations in asthma risk by finely defined age-groups. In so doing, we extend similar Canadian work conducted in Toronto [14], Vancouver [15], and Saint John [16]. To date, associations between air pollution and hospital visits for asthma have not been examined in the Canadian province of Alberta. Moreover, only a few studies [17-20] have examined the extent through which exposure to aeroallergens may confound associations between outdoor air pollution and asthma ED visits.

## Methods

### Hospital emergency department visit data

Anonymous patient data were provided by Capital Health (CH), a public sector organization funded by the province of Alberta that provides health service to all individuals (approximately one million) located in the census metropolitan area of Edmonton, Alberta. CH also provides specialized services such as trauma and burn treatment, organ transplants, and high risk obstetrics for a larger catchment population of 1.6 million persons in central and northern Alberta [21]. ED visit data used in this study were provided by five hospitals that have a full service ED with in-patient beds and provide 24 hour service. While there are other EDs in operation in the Edmonton area, they service less than 20% of the ED visits in a given year. These hospitals are staffed by full-time emergency physicians. Each ED visit is coded by experienced medical record nosologists using the triage information, nursing notes, ED records and consultation notes. ED department visits were classified according to the International Classification for diseases 9^th ^revision (ICD-9) based on the discharge diagnosis.

Available patient data allowed us to examine the relationship between air pollution and ED visits for asthma between April 1, 1992 and March 31, 2002. Additional information contained in the database, and used in this study, included the date of visit, and the age and sex of the patient. While we restricted our analyses to visits for asthma (ICD-9: 493), we also tabulated the number of daily visits for influenza (ICD-9: 487). This allowed us to adjust our air pollution risk estimates for asthma for the possible confounding influence of viral respiratory seasonal epidemics.

We excluded visits among infants less than two years of age as the diagnosis of asthma in this age range can prove problematic. In total, there were 58,888 ED visits for asthma observed over the study period. For 86% of these visits, a unique patient identification number was available. Using this variable, we were able to identify multiple visits on the same day by the same patient. Only one ED visit per person was included in our analyses resulting in an exclusion of 976 observations. Therefore, the risk estimates presented in this paper are based on a total of 57,912 ED visits.

### Air pollution and meteorological data

Daily air pollution levels were obtained from automated fixed-site monitoring stations maintained by Environment Canada as part of the National Air Pollution Surveillance Network [22]. The daily means were calculated as the average of 24 hourly measures in the same day; daily pollution levels were considered missing if any of the 24 hourly measures were not available. Data were obtained for carbon monoxide (CO), nitrogen dioxide (NO_2_), ozone (O_3_), sulfur dioxide (SO_2_), and particulate matter of median aerometric diameter less than 10 and 2.5 microns (PM_10_, PM_2.5 _respectively). CO, NO_2_, O_3 _and SO_2 _were measured using "reference methods" or "equivalent methods" as designated by the United States Environmental Protection Agency. CO was measured using non-dispersive infrared spectrometry, NO_2 _using chemiluminesence, O_3 _using chemiluminesence/ultraviolet photometry and SO_2 _using coulometry/ultraviolet fluorescence. PM_2.5 _and PM_10 _were measured using tapered element oscillating microbalance (TEOM) instruments; however, they were not routinely monitored until 1998. Daily data were averaged across the 3 monitoring stations that were in operation during the study interval. Environment Canada also provided meteorological data from the monitoring station at the Edmonton airport. These included daily mean temperature and relative humidity which were used as adjustment factors in our multivariable conditional logistic regression models.

### Aeroallergen data

Pollen grains and fungal spore data were collected by using rotational impaction sampling methods. Particles that adhered to the silicone grease-coated sampling rods were analyzed to determine the number of particles per cubic meter of air sampled in a 24 hour period. Similar to previous analyses using related data [23], we calculated daily levels for Basidiomycetes, Ascomycetes, Deuteromycetes, and weeds, trees, and grass pollen.

### Statistical methods

All statistical analyses were done using SAS [24]. Associations of asthma visits with outdoor air pollution were formally investigated using statistical methods appropriate for the case-crossover study design. This design is an adaptation of the case-control study in which cases serve as their own controls [25]. For each ED visit, an individual's exposure at the "index" time was compared to their exposure at a referent time interval. Because within-individual comparisons are being made, there is no confounding due to time-independent risk factors. By selecting referent intervals that are close in time to the case event, seasonal patterns in disease occurrence are controlled for. Similarly, the matching of control to case periods by day of week ostensibly controls for the influence of "day of week" effects on the frequency of ED visits. In our study, the case period refers to the day that the ED visit for asthma occurred.

While referent periods are individually matched to case periods, case-crossover studies have used several different strategies to select them. The implications of these selection methods on risk estimates have recently been evaluated in great detail [26]. Based on this work, we chose our referent periods by using a time-stratified design. Specifically, referents were selected from the same day of the week, month and year as the case interval. This approach to the selection of referent intervals is not subject to time trend biases, and ensures unbiased conditional logistic regression estimates [26]. Once these matched sets consisting of one case period and either three or four referent periods had been assembled, conditional logistic regression was used to produce the risk estimates. These were represented by the odds ratios (OR), and the accompanying 95% confidence intervals were used to assess statistical significance. The SAS procedure PHREG [24] was used to perform these analyses. Similar analyses were undertaken to examine whether associations between air pollution and asthma were similar across age-groups, between men and women, and by season (April to September, October to March). These months were selected to classify seasons for several reasons. First, from the perspective of statistical power, this dichotomy produced a nearly equal number of ED visits in both periods. Second, the period April to September represents the period where individuals spend a greater portion of their time outdoors, and air pollution levels estimated from fixed-site monitoring stations may better reflect their true exposure. Lastly, during this spring/summer period, individuals are also more likely to be exposed to aeroallergens.

*A priori*, we constructed several different metrics to examine the temporal relationship between outdoor air pollution levels and the time when an individual presented to an ED for asthma. These metrics included: the same day exposure, 1, 2 and 3 day lagged exposures, as well as cumulative 3-day and 5-day mean exposure estimates. For all air pollutants, with the exception of ozone, daily mean exposure estimates were used. Ozone values were based on the 8-hour maximum value.

For meteorological time-varying covariates of temperature and relative humidity we evaluated their potential confounding role as lagged (0, 1, 2 day) or cumulative exposures (3, 5 day average), and as linear and quadratic terms. For the most part, there were no appreciable differences in risk estimates for more complex meteorological adjustments, and therefore, our risk estimates are adjusted for linear same day effects of temperature and relative humidity.

Our risk estimates were derived primarily by using single pollutant models. As is commonly done, for each pollutant, we have expressed our odds ratios according to an increase in the interquartile range (IQR). The IQR was calculated based on the daily mean levels of each air pollutant over the entirety of the study period. Two pollutant models were also fit to evaluate how positive associations, noted for some pollutants, changed after adjusting for daily levels of other pollutants.

## Results

**Figure 1:**
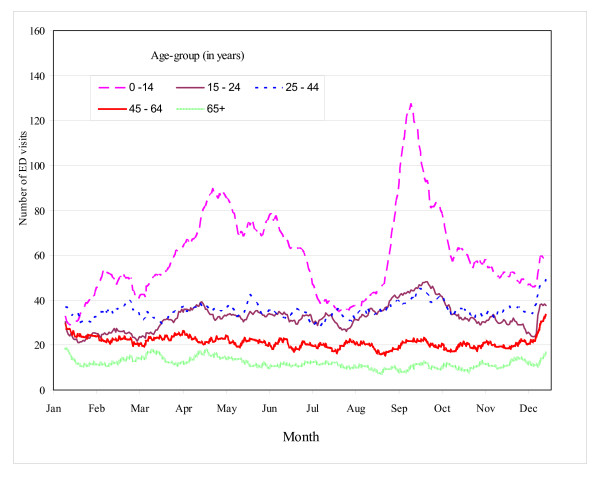
**Number of asthma emergency department visits in Edmonton**. Number of emergency department visits for asthma by month and age-group, Edmonton, April 1, 1992 to March 31, 2002.

Asthma visits accounted for approximately 2% of all ED visits in these 5 hospitals. A total of 57,912 emergency department visits were identified during the study interval and formed the basis of the case-crossover analyses (Table 1). Almost 13% of these visits occurred in young children (2 – 4 years of age), while 8.1% occurred among those aged 65 years of age and older. There were slightly more visits among females (51.8%) than their male counterparts. A total of 30,576 visits occurred between the months of April to September. Trends in the frequency of asthma ED visits over the course of the year, by age group, are presented in Figure 1. A peak was observed in late September; this excess was most pronounced among children.

**Table 1:** Number of emergency department visits for asthma, by age-group, sex, and season

**Characteristic**	**Number of visits**	**%**
**Age (in years)**		
2 – 4	7,247	12.5
5 – 14	13,145	22.7
15 – 24	11,616	20.1
25 – 44	13,300	23.0
45 – 64	7,899	13.6
65 – 74	2,850	4.9
≥ 75	1,855	3.2
Sex		
Male	27,926	48.2
Female	29,986	51.8
Season		
Spring/Summer (April to September)	30,576	52.8
Fall/Winter (October to March)	27,336	47.2

Total visits	57,912	100.0

Daily levels of ambient air pollution over the study period are presented in Table 2. Gaseous pollutant data were available for the entire study period (1992 to 2002), while daily particulate matter data were only available from 1998 onwards. For the most part, pollution levels were higher during the winter season, the notable exception being ozone. Aeroallergen levels for the period 1996 to 2002 are also described. For the most part, no sampling for aeroallergens was completed during the winter season since pollen, during the majority of that period, would be absent or at a very low level and would not be significant for patient reactions. Regarding the fungal spores, it is expected that levels would be insignificant during the months from December to beginning of collections and although levels during late fall may be significant, the data remains unavailable. Pearson correlation coefficients were generated to better understand the associations between pollutants and aeroallergens [data not shown]. NO_2 _and CO were strongly correlated with each other (r = 0.74), as were PM_2.5 _and PM_10 _(r = 0.79). Ozone was strongly correlated with temperature and relative humidity (r = 0.54). The strongest associations between pollutant and aeroallergen levels were observed with Ascomycetes and NO_2 _(r = -0.26), pollens from trees and O_3 _(daily max) (r = 0.24), Ascomycetes and O_3 _(daily max) (r = -0.16).

**Table 2:** Frequency distribution of the daily pollution levels for gaseous and particulate phase pollutants

	**Summer (April to September)**				**Winter (October to March)**			
	
	Days*	25^th ^P	75^th ^P	Med	Days*	25^th ^P	75^th ^P	Med
SO_2 _(ppb)	1830	1.0	3.0	2.0	1822	2.0	4.0	3.0
NO_2 _(ppb)	1830	14.0	22.0	17.5	1822	22.5	35.5	28.5
CO (ppm)	1830	0.5	0.7	0.6	1822	0.7	1.3	0.9
O_3- _daily max (ppb)	1830	29.5	46.0	38.0	1822	16.5	31.5	24.3
PM_2.5: _g/m^3^	715	4.5	11.0	7.0	729	5.0	11.0	7.3
PM_10: _g/m^3^	732	15.0	32.5	22.0	819	13.0	29.0	19.0
Temperature (°C)	1830	9.8	17.2	13.9	1822	-11.1	1.6	-3.8
Relative humidity (%)	1830	53.0	72.8	63.0	1822	60.9	78.0	68.8
*Aeroallergens*†								
Grasses (spores/m^3^)	1098	0.0	5.7	1.0	1093	0.0	0.0	0.0
Trees (spores/m^3^)	1098	0.0	42.2	3.4	1093	0.0	0.0	0.0
Weeds (grains/m3)	1098	0.0	5.0	0.0	1093	0.0	0.0	0.0
Deuteromycetes (spores/m^3^)	1098	350.4	2303.7	1096.9	1093	0.0	0.0	0.0
Basidiomycetes(spores/m^3^)	1098	19.1	442.8	124.8	1093	0.0	0.0	0.0
Asomycetes (spores/m^3^)	1098	59.1	519.4	194.2	1093	0.0	0.0	0.0

* Number of days with non-missing values;

≸Aeroallergen data available from 1996 to 2002

Adjusted odds ratios for ED visits for asthma according to levels of ambient air pollution, by season, are displayed in Table 3. For the most part, there were no statistically significant associations between air pollution levels and ED visits for asthma in the period between October and March (fall/winter). In contrast, statistically significant associations were observed in the period between April and September with NO_2_, CO, PM_2.5_, PM_10 _and O_3_. These associations were stronger when the 5-day average was used, relative to the other shorter term exposures indices that we used. No association was evident for SO_2_.

**Table 3:** Adjusted odds ratios* for emergency department visits for asthma, patients of all ages, by season

Pollutant	Mean	IQR	**All seasons**		**Season**			
					
					**October to March**		**April to September**	
			
			**OR**	**95% C.I**.	**OR**	**95% C.I**.	**OR**	**95% C.I**.
SO_2_	Same day	3.0	0.97	0.95–0.98	0.96	0.93–0.98	0.98	0.95–1.00
	1-day lag		0.98	0.96–0.99	0.96	0.94–0.98	1.00	0.97–1.02
	3-day average		0.95	0.93–0.98	0.93	0.90–0.96	0.98	0.94–1.02
	5-day average		0.95	0.93–0.98	0.93	0.90–0.97	0.98	0.93–1.02
NO_2_	Same day	13.5	0.99	0.97–1.01	0.98	0.96–1.00	1.01	0.98–1.05
	1-day lag		1.01	0.99–1.03	0.99	0.97–1.01	1.07	1.03–1.10
	3-day average		1.01	0.99–1.03	0.98	0.95–1.00	1.09	1.04–1.13
	5-day average		1.03	1.00–1.05	0.98	0.95–1.01	1.14	1.09–1.20
CO	Same day	0.5	0.99	0.98–1.00	0.98	0.97–0.99	1.04	1.01–1.08
	1-day lag		1.00	0.99–1.02	0.99	0.98–1.01	1.06	1.02–1.10
	3-day average		1.00	0.99–1.02	0.98	0.97–1.00	1.11	1.06–1.16
	5-day average		1.02	1.00–1.04	0.99	0.97–1.01	1.18	1.11–1.25
O_3 _(Max)	Same day	18.0	1.02	1.00–1.04	1.02	0.98–1.05	1.04	1.01–1.07
	1-day lag		1.04	1.02–1.06	1.02	0.99–1.06	1.06	1.04–1.09
	3-day average		1.07	1.04–1.10	1.05	1.00–1.10	1.11	1.07–1.16
	5-day average		1.08	1.05–1.11	1.07	1.02–1.13	1.11	1.06–1.15
PM_2.5_^‡^	Same day	6.3	1.04	1.02–1.05	1.00	0.97–1.03	1.07	1.05–1.09
	1-day lag		1.01	1.00–1.03	0.99	0.97–1.02	1.03	1.01–1.05
	3-day average		1.04	1.02–1.06	0.99	0.96–1.03	1.08	1.05–1.11
	5-day average		1.04	1.01–1.06	0.98	0.94–1.02	1.08	1.05–1.12
PM_10_^‡^	Same day	16.0	1.04	1.02–1.06	1.00	0.98–1.03	1.07	1.04–1.09
	1-day lag		1.02	1.01–1.04	1.02	0.99–1.05	1.03	1.01–1.05
	3-day average		1.05	1.02–1.07	1.01	0.98–1.05	1.08	1.05–1.11
	5-day average		1.04	1.02–1.07	1.01	0.97–1.05	1.08	1.04–1.12

*Odds ratios were calculated in relation to an increase in the interquartile range (IQR) of selected air pollutants and were adjusted for relative humidity and temperature, and daily number of visits for influenza (all ages combined).

^‡ ^Particulate data were only available between January 1, 1998 and December 31, 2002

In Tables 4, 5, 6, 7, 8, 9, analyses are replicated for different age groups. Associations between air pollution and ED visits for asthma were consistently found in the summer/spring season and absent in the fall/winter. Therefore, the discussion of results in this paragraph is limited to our findings for the April to September period. The most marked associations with ED visits and air pollution levels occurred among very young children (2 – 4 years of age) (Table 4). In this age group, an increase in the interquartile range of the 5-day average for NO_2 _or CO was associated with 50% and 48% increases, respectively, in the risk of an asthma ED visit (p < 0.05). No association was observed with SO_2_, however, increased levels of O_3_, PM_2.5 _and PM_10 _were positively associated with the number of ED visits for asthma (p < 0.05).

**Table 4:** Adjusted odds ratios* for emergency department visits for asthma among individuals 2 – 4 years of age, by season

Pollutant	Mean	IQR	All seasons		Season			
					
					October to March		April to September	
			
			OR	95% C.I.	OR	95% C.I.	OR	95% C.I.
SO_2_	Same day	3.0	1.00	0.95–1.05	0.98	0.92–1.05	1.01	0.94–1.08
	1-day lag		0.95	0.90–1.00	0.93	0.87–0.99	0.95	0.89–1.03
	3-day average		0.97	0.90–1.04	0.93	0.85–1.02	0.99	0.89–1.11
	5-day average		0.96	0.89–1.04	0.91	0.82–1.02	0.99	0.87–1.12
NO_2_	Same day	13.5	1.00	0.95–1.05	0.96	0.91–1.02	1.08	0.99–1.18
	1-day lag		1.04	0.99–1.09	0.96	0.90–1.02	1.24	1.13–1.35
	3-day average		1.05	0.98–1.12	0.95	0.88–1.02	1.32	1.18–1.48
	5-day average		1.08	1.00–1.16	0.93	0.85–1.01	1.50	1.31–1.71
CO	Same day	0.5	0.99	0.95–1.03	0.97	0.93–1.01	1.06	0.97–1.15
	1-day lag		1.02	0.98–1.05	0.99	0.95–1.03	1.14	1.04–1.25
	3-day average		1.02	0.98–1.07	0.98	0.93–1.03	1.25	1.11–1.42
	5-day average		1.04	0.98–1.10	0.97	0.91–1.03	1.48	1.27–1.72
O_3 _(Max)	Same day	18.0	1.00	0.94–1.07	1.04	0.94–1.14	1.02	0.93–1.12
	1-day lag		1.03	0.97–1.10	1.08	0.98–1.18	1.05	0.97–1.14
	3-day average		1.06	0.97–1.14	1.12	0.99–1.27	1.10	0.98–1.22
	5-day average		1.06	0.97–1.15	1.16	1.01–1.34	1.06	0.94–1.19
PM_2.5_^‡^	Same day	6.3	1.02	0.96–1.07	1.02	0.94–1.11	1.06	0.99–1.14
	1-day lag		1.02	0.97–1.08	0.98	0.91–1.07	1.08	1.01–1.16
	3-day average		1.04	0.98–1.12	1.00	0.91–1.11	1.15	1.05–1.26
	5-day average		1.03	0.95–1.11	0.95	0.84–1.07	1.16	1.04–1.28
PM_10_^‡^	Same day	16.0	1.05	0.99–1.10	1.04	0.95–1.13	1.10	1.02–1.18
	1-day lag		1.03	0.97–1.08	1.00	0.92–1.09	1.07	1.00–1.15
	3-day average		1.07	1.00–1.14	1.02	0.92–1.13	1.14	1.04–1.26
	5-day average		1.07	0.99–1.16	1.00	0.89–1.13	1.16	1.05–1.28

*Odds ratios were calculated in relation to an increase in the interquartile range (IQR) of selected air pollutants and were adjusted for relative humidity and temperature, and daily number of visits for influenza (all ages combined).

^‡ ^Particulate data were only available between January 1, 1998 and December 31, 2002

**Table 5:** Adjusted odds ratios* for emergency department visits for asthma among patients 5 – 14 years of age, by season

Pollutant	Mean	IQR	All seasons		Season			
					
					October to March		April to September	
			
			OR	95% C.I.	OR	95% C.I.	OR	95% C.I.
SO_2_	Same day	3.0	0.96	0.93–1.00	0.98	0.93–1.03	0.94	0.89–0.99
	1-day lag		0.98	0.94–1.02	0.98	0.93–1.03	0.97	0.93–1.03
	3-day average		0.94	0.90–1.00	0.96	0.89–1.03	0.92	0.85–1.00
	5-day average		0.97	0.91–1.03	1.01	0.93–1.10	0.91	0.83–1.00
NO_2_	Same day	13.5	0.99	0.95–1.03	0.98	0.93–1.03	1.00	0.94–1.06
	1-day lag		1.05	1.01–1.09	1.04	0.99–1.09	1.08	1.01–1.15
	3-day average		1.05	1.00–1.10	1.03	0.97–1.10	1.08	0.99–1.17
	5-day average		1.09	1.03–1.15	1.07	1.00–1.15	1.13	1.02–1.24
CO	Same day	0.5	0.98	0.96–1.01	0.97	0.94–1.00	1.02	0.95–1.08
	1-day lag		1.03	1.00–1.05	1.02	0.99–1.05	1.06	1.00–1.14
	3-day average		1.02	0.98–1.05	1.01	0.97–1.05	1.05	0.96–1.15
	5-day average		1.05	1.01–1.09	1.04	1.00–1.09	1.09	0.98–1.22
O_3 _(Max)	Same day	18.0	1.02	0.97–1.07	1.00	0.92–1.07	1.05	0.98–1.12
	1-day lag		1.05	1.00–1.10	0.99	0.92–1.06	1.09	1.03–1.16
	3-day average		1.10	1.03–1.17	1.02	0.93–1.13	1.16	1.07–1.25
	5-day average		1.10	1.03–1.17	1.04	0.93–1.16	1.14	1.05–1.24
PM_2.5_^‡^	Same day	6.3	1.04	1.00–1.07	1.02	0.96–1.08	1.04	1.00–1.09
	1-day lag		1.02	0.98–1.06	1.00	0.94–1.06	1.03	0.98–1.07
	3-day average		1.05	1.00–1.10	1.01	0.93–1.09	1.07	1.01–1.14
	5-day average		1.06	1.00–1.12	0.99	0.91–1.09	1.10	1.02–1.17
PM_10_^‡^	Same day	16.0	1.06	1.02–1.10	1.04	0.98–1.11	1.07	1.02–1.13
	1-day lag		1.04	1.00–1.08	1.02	0.96–1.09	1.04	1.00–1.09
	3-day average		1.08	1.03–1.14	1.03	0.96–1.12	1.11	1.05–1.18
	5-day average		1.09	1.03–1.15	1.02	0.93–1.11	1.14	1.06–1.22

*Odds ratios were calculated in relation to an increase in the interquartile range (IQR) of selected air pollutants and were adjusted for relative humidity and temperature, and daily number of visits for influenza (all ages combined).

^‡ ^Particulate data were only available between January 1, 1998 and December 31, 2002

**Table 6:** Adjusted odds ratios* for emergency department visits for asthma among patients 15 – 44 years of age, by season

Pollutant	Mean	IQR	All seasons		Season			
					
					October to March		April to September	
			
			OR	95% C.I.	OR	95% C.I.	OR	95% C.I.
SO_2_	Same day	3.0	0.96	0.93–0.98	0.93	0.90–0.97	0.98	0.94–1.02
	1-day lag		0.98	0.96–1.01	0.95	0.92–0.98	1.02	0.98–1.06
	3-day average		0.95	0.91–0.98	0.90	0.86–0.95	1.00	0.94–1.06
	5-day average		0.93	0.89–0.97	0.88	0.83–0.93	1.01	0.94–1.08
NO_2_	Same day	13.5	0.98	0.96–1.01	0.96	0.93–1.00	1.04	0.99–1.09
	1-day lag		0.99	0.97–1.02	0.97	0.94–1.01	1.04	0.99–1.09
	3-day average		0.98	0.95–1.02	0.95	0.91–0.99	1.07	1.00–1.14
	5-day average		0.98	0.95–1.02	0.94	0.90–0.99	1.10	1.02–1.19
CO	Same day	0.5	0.99	0.97–1.01	0.97	0.95–1.00	1.09	1.03–1.14
	1-day lag		1.00	0.98–1.02	0.99	0.97–1.01	1.04	0.99–1.10
	3-day average		0.99	0.97–1.02	0.97	0.94–1.00	1.14	1.06–1.23
	5-day average		1.00	0.97–1.03	0.97	0.94–1.00	1.20	1.10–1.31
O_3 _(Max)	Same day	18.0	1.03	1.00–1.07	1.04	0.99–1.09	1.05	1.00–1.10
	1-day lag		1.04	1.01–1.07	1.03	0.98–1.08	1.07	1.03–1.12
	3-day average		1.07	1.03–1.12	1.06	0.99–1.14	1.11	1.05–1.18
	5-day average		1.08	1.03–1.13	1.09	1.01–1.17	1.11	1.04–1.18
PM_2.5_^‡^	Same day	6.3	1.04	1.02–1.06	0.99	0.95–1.03	1.07	1.04–1.10
	1-day lag		1.00	0.98–1.02	0.99	0.95–1.03	1.01	0.98–1.04
	3-day average		1.02	0.99–1.05	0.98	0.93–1.03	1.06	1.02–1.10
	5-day average		1.01	0.97–1.04	0.96	0.90–1.02	1.05	1.00–1.10
PM_10_^‡^	Same day	16.0	1.02	0.99–1.05	0.99	0.95–1.03	1.05	1.02–1.09
	1-day lag		1.01	0.98–1.03	1.02	0.98–1.07	1.00	0.96–1.03
	3-day average		1.02	0.99–1.05	1.01	0.96–1.06	1.03	0.99–1.08
	5-day average		1.00	0.97–1.04	0.99	0.94–1.05	1.02	0.97–1.07

*Odds ratios were calculated in relation to an increase in the interquartile range (IQR) of selected air pollutants and were adjusted for relative humidity and temperature, and daily number of visits for influenza (all ages combined).

^‡ ^Particulate data were only available between January 1, 1998 and December 31, 2002

**Table 7:** Adjusted odds ratios* for emergency department visits for asthma among patients 45 – 64 years of age, by season

Pollutant	Mean	IQR	All seasons		Season			
					
					October to March		April to September	
			
			OR	95% C.I.	OR	95% C.I.	OR	95% C.I.
SO_2_	Same day	3.0	0.97	0.92–1.01	0.96	0.91–1.02	0.96	0.90–1.04
	1-day lag		0.99	0.95–1.04	0.99	0.94–1.05	0.99	0.93–1.07
	3-day average		0.95	0.89–1.02	0.94	0.87–1.02	0.98	0.88–1.09
	5-day average		0.96	0.89–1.04	0.96	0.87–1.05	0.98	0.86–1.12
NO_2_	Same day	13.5	0.97	0.92–1.01	0.99	0.94–1.04	0.89	0.81–0.98
	1-day lag		0.98	0.93–1.02	0.97	0.92–1.03	0.98	0.89–1.08
	3-day average		0.95	0.89–1.00	0.95	0.89–1.02	0.93	0.82–1.04
	5-day average		0.96	0.89–1.02	0.95	0.88–1.03	0.99	0.86–1.14
CO	Same day	0.5	1.00	0.97–1.03	1.01	0.97–1.05	0.93	0.84–1.03
	1-day lag		0.99	0.96–1.03	0.99	0.95–1.03	1.04	0.94–1.15
	3-day average		0.98	0.94–1.03	0.99	0.94–1.04	0.96	0.84–1.11
	5-day average		0.98	0.93–1.03	0.99	0.93–1.04	1.00	0.84–1.18
O_3 _(Max)	Same day	18.0	1.00	0.94–1.06	0.97	0.89–1.06	1.01	0.92–1.10
	1-day lag		1.05	1.00–1.11	1.04	0.96–1.13	1.04	0.97–1.13
	3-day average		1.08	1.00–1.16	1.06	0.94–1.18	1.07	0.97–1.19
	5-day average		1.12	1.03–1.22	1.09	0.96–1.24	1.12	1.00–1.26
PM_2.5_^‡^	Same day	6.3	1.04	1.00–1.08	0.96	0.90–1.03	1.08	1.03–1.14
	1-day lag		1.04	1.00–1.08	1.00	0.93–1.07	1.06	1.01–1.11
	3-day average		1.06	1.01–1.12	0.97	0.89–1.07	1.12	1.05–1.20
	5-day average		1.07	1.01–1.14	0.98	0.88–1.09	1.14	1.05–1.23
PM_10_^‡^	Same day	16.0	1.04	0.99–1.09	1.00	0.93–1.07	1.08	1.02–1.15
	1-day lag		1.05	1.01–1.10	1.02	0.95–1.10	1.07	1.01–1.14
	3-day average		1.06	1.00–1.12	0.99	0.91–1.08	1.12	1.04–1.22
	5-day average		1.06	0.99–1.13	0.99	0.90–1.10	1.13	1.03–1.23

*Odds ratios were calculated in relation to an increase in the interquartile range (IQR) of selected air pollutants and were adjusted for relative humidity and temperature, and daily number of visits for influenza (all ages combined).

^‡ ^Particulate data were only available between January 1, 1998 and December 31, 2002

**Table 8:** Adjusted odds ratios* for emergency department visits for asthma among patients 65 – 74 years of age, by season

Pollutant	Mean	IQR	All seasons		Season			
					
					October to March		April to September	
			
			OR	95% C.I.	OR	95% C.I.	OR	95% C.I.
SO_2_	Same day	3.0	1.01	0.94–1.09	1.01	0.91–1.11	1.00	0.89–1.13
	1-day lag		0.94	0.87–1.01	0.92	0.84–1.01	0.95	0.84–1.07
	3-day average		0.98	0.87–1.09	0.95	0.83–1.09	1.00	0.83–1.21
	5-day average		0.97	0.86–1.11	0.97	0.83–1.14	0.95	0.76–1.18
NO_2_	Same day	13.5	1.03	0.96–1.11	1.04	0.96–1.14	0.99	0.86–1.16
	1-day lag		0.99	0.92–1.07	0.99	0.90–1.08	1.00	0.86–1.16
	3-day average		1.05	0.96–1.16	1.05	0.94–1.17	1.06	0.88–1.29
	5-day average		1.09	0.97–1.21	1.07	0.94–1.22	1.12	0.89–1.41
CO	Same day	0.5	1.01	0.96–1.07	1.01	0.95–1.07	1.03	0.88–1.21
	1-day lag		0.98	0.92–1.04	0.97	0.92–1.04	1.00	0.84–1.19
	3-day average		1.01	0.94–1.09	1.00	0.93–1.09	1.03	0.82–1.29
	5-day average		1.02	0.94–1.11	1.01	0.93–1.11	1.02	0.78–1.35
O_3_	Same day	18.0	0.96	0.87–1.07	0.94	0.81–1.09	1.01	0.87–1.18
	1-day lag		1.03	0.94–1.13	1.03	0.90–1.18	1.05	0.92–1.20
	3-day average		0.99	0.88–1.13	0.92	0.76–1.10	1.12	0.93–1.34
	5-day average		1.00	0.87–1.15	0.90	0.73–1.11	1.14	0.94–1.39
PM_2.5_^‡^	Same day	6.3	1.04	0.97–1.12	1.04	0.92–1.18	1.06	0.96–1.16
	1-day lag		1.00	0.92–1.08	0.99	0.88–1.11	1.01	0.91–1.11
	3-day average		1.06	0.97–1.17	1.08	0.93–1.26	1.06	0.94–1.20
	5-day average		1.10	0.98–1.23	1.12	0.94–1.34	1.10	0.95–1.27
PM_10_^‡^	Same day	16.0	1.06	0.97–1.15	1.04	0.92–1.17	1.10	0.98–1.23
	1-day lag		1.00	0.92–1.09	0.98	0.87–1.11	1.03	0.91–1.15
	3-day average		1.08	0.97–1.19	1.06	0.91–1.23	1.11	0.96–1.27
	5-day average		1.10	0.98–1.24	1.10	0.92–1.31	1.12	0.95–1.32

*Odds ratios were calculated in relation to an increase in the interquartile range (IQR) of selected air pollutants and were adjusted for relative humidity and temperature, and daily number of visits for influenza (all ages combined).

^‡ ^Particulate data were only available between January 1, 1998 and December 31, 2002

**Table 9:** Adjusted odds ratios* for emergency department visits for asthma among patients 75 years of age and older, by season

Pollutant	Mean	IQR	All seasons		Season			
					
					October to March		April to September	
			
			OR	95% C.I.	OR	95% C.I.	OR	95% C.I.
SO_2_	Same day	3.0	0.96	0.88–1.06	0.93	0.82–1.05	1.01	0.87–1.17
	1-day lag		1.01	0.92–1.11	0.99	0.88–1.12	1.03	0.89–1.19
	3-day average		1.03	0.90–1.18	0.99	0.84–1.18	1.09	0.87–1.38
	5-day average		1.06	0.90–1.24	1.04	0.86–1.27	1.07	0.81–1.42
NO_2_	Same day	13.5	1.00	0.91–1.10	0.96	0.86–1.07	1.14	0.94–1.37
	1-day lag		1.09	0.99–1.20	1.08	0.97–1.21	1.13	0.93–1.36
	3-day average		1.13	1.00–1.27	1.07	0.93–1.24	1.33	1.03–1.70
	5-day average		1.20	1.04–1.38	1.15	0.98–1.35	1.37	1.02–1.84
CO	Same day	0.5	0.96	0.89–1.03	0.94	0.87–1.01	1.11	0.91–1.35
	1-day lag		1.01	0.94–1.09	1.00	0.93–1.08	1.12	0.91–1.37
	3-day average		1.00	0.91–1.10	0.97	0.88–1.07	1.28	0.96–1.69
	5-day average		1.08	0.97–1.20	1.04	0.93–1.16	1.54	1.09–2.17
O_3_	Same day	18.0	1.08	0.96–1.23	1.07	0.90–1.29	1.10	0.92–1.32
	1-day lag		0.98	0.87–1.10	0.97	0.82–1.16	0.99	0.84–1.16
	3-day average		1.02	0.87–1.19	1.00	0.79–1.27	1.04	0.84–1.30
	5-day average		1.01	0.85–1.20	1.04	0.80–1.36	0.99	0.78–1.25
PM_2.5_^‡^	Same day	6.3	1.12	1.01–1.23	1.06	0.92–1.22	1.19	1.02–1.40
	1-day lag		1.06	0.96–1.16	1.02	0.89–1.17	1.08	0.94–1.23
	3-day average		1.11	0.98–1.26	1.05	0.87–1.26	1.16	0.96–1.41
	5-day average		1.13	0.97–1.30	1.16	0.94–1.44	1.07	0.86–1.33
PM_10_^‡^	Same day	16.0	1.06	0.95–1.17	0.96	0.82–1.12	1.17	1.00–1.36
	1-day lag		1.06	0.96–1.17	1.03	0.90–1.18	1.10	0.95–1.26
	3-day average		1.04	0.92–1.18	0.96	0.80–1.14	1.14	0.95–1.37
	5-day average		1.07	0.92–1.23	1.08	0.89–1.32	1.06	0.86–1.30

*Odds ratios were calculated in relation to an increase in the interquartile range (IQR) of selected air pollutants and were adjusted for relative humidity and temperature, and daily number of visits for influenza (all ages combined).

^‡ ^Particulate data were only available between January 1, 1998 and December 31, 2002

**Figure 2:**
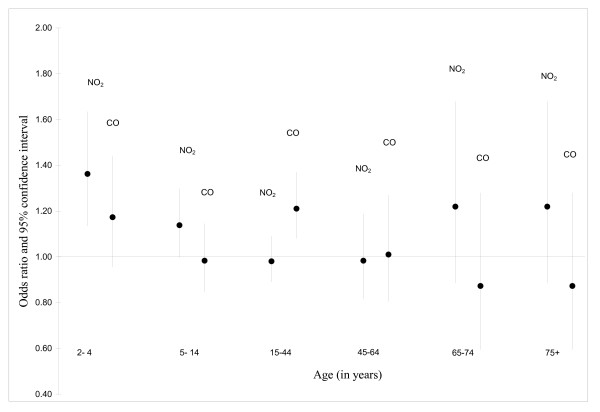
**Associations between asthma visits and levels of NO2 and CO**. Adjusted odds ratios obtained from a two-pollutant model in relation to an increase in the interquartile range of 5-day average concentration of NO_2 _and CO, Edmonton, April 1 to September 30, 1992 to 2002; Adjusted for relative humidity, temperature and daily number of emergency department visits for influenza.

NO_2 _and PM_10 _were the pollutants for which the strongest associations were observed among children 5–14 years of age (Table 5). In general, associations were strongest for the air pollution metric constructed using the 5-day average. Among those aged 15–44, associations were less pronounced than those found in children, however, NO_2 _and CO signals were evident (Table 6). Relative to the findings for other adults, associations were stronger among those 75 years of age and older (Table 9). Specifically, an increase in the interquartile range of the 5-day average for O_3 _was associated with a 54% increase in the number of asthma visits in this age group; the corresponding estimate for NO_2 _was 37%. Two pollutant modeling revealed stronger associations for NO_2_, relative to CO, for all age groups except those aged 15 – 44 years of age (Figure 2).

The addition of aeroallergens to the age-specific model did not produce a material change in the air pollution risk estimates shown in Table 4 to 9. Like air pollution levels, the magnitude of the aeroallergen derived odds ratios was strongest for the 5-day average exposure index. However, the effects were much smaller in magnitude than those found for changes in air pollution levels. The strongest association was found for Deuteromycetes where an IQR change in the 5-day average resulted in an odds ratio of 1.03 (95% CI = 1.00–1.06) for an ED visit for asthma.

## Discussion

The economic burden of asthma is considerable, and therefore, it is valuable to identify exposures that can be modified on a population-level basis to reduce the health care costs to treat it. In 1994, the economic costs of asthma for the US were estimated to be in excess of $10 billion dollars [27]. The corresponding estimate for Canada, in 1990, was estimated to fall in the range between $504 and $648 million [28]. A review of published studies found that hospital costs typically account for 20–25% of the overall direct costs of asthma [29]. Given that there are approximately 150,000 ED visits for asthma in Canada annually, a modest reduction in these numbers alone would provide considerable costs saving. We found positive associations between outdoor levels of air pollution and asthma ED visits, between April and September, in each age-group examined. Associations were generally stronger for NO_2 _and CO, however, they were also evident for O_3 _and particulate matter. These findings provide compelling evidence that reductions in emissions from the sources that give rise to these pollutant levels may decrease the associated direct health care costs of asthma in this region. Elsewhere, interventions to reduce outdoor air pollution levels have proven to be successful as they were accompanied with a concomitant decrease in the number of hospital visits and admissions for asthma, particularly in young children [30,31].

This study was undertaken, in part, because there have been few Canadian studies that have evaluated how associations between ambient levels of air pollution and hospitals visits for asthma vary by age. In addition, the composition mix of pollution in Edmonton differs from other Canadian cities due to the close proximity of coal and petrochemical industries. The findings of our study are similar to those reported for a case-crossover study in Toronto where positive associations with CO and NO_2 _and hospital admissions for asthma in both males and females aged 6 to 12 were observed, however no effect was found for O_3 _[14]. Further support for the relevance of vehicular traffic on asthma comes from the work by Oyana who reported an increased prevalence of asthma among children and adults who lived in close proximity to ambient sources of pollution at a US-Canada border crossing [32,33]. In contrast, associations with ozone have been noted in the Canadian cities of Saint John, New Brunswick [34], but not with NO_2 _[16]. Positive associations with ozone have been also noted in a study of ED visits in the province of Ontario and Toronto [35,36]; for the province wide study, effects were more pronounced among children under the age of one, however, asthma remains an unclear diagnosis in children under the age of two.

Positive associations were observed with both particulate and gaseous phase pollution and as previously mentioned, they were most evident with NO_2 _and CO, both typically regarded as markers of vehicular traffic. In the province of Alberta, transportation accounts for a much smaller percentage of overall nitrogen oxides (NO_X_) emissions (26%), than it does in Canada as a whole (50%) [37]. Therefore, it is possible that industrial sources of NO_2 _in the Edmonton area contribute to the increased risk of asthma visits. The nature of the hospital or pollution data does not allow us to evaluate the respective contributions of industrial versus transportation sources of pollution to the increased risk of asthma visits.

To better understand the interrelationship between CO, NO_2 _which were highly correlated with each other (r = 0.70), two-pollutant models were fit. These analyses revealed stronger associations with ED asthma visits among children and the elderly for NO_2_, relative to CO, in all age ranges except among those aged 15 to 45. There exist several biological mechanisms whereby NO_2 _can affect respiratory health. It has been shown to make people more susceptible to respiratory viral infections that exacerbate asthma [38], and enhance allergic responses after subsequent challenge [39]. NO_2 _has also been shown to increase bronchitis symptoms among asthmatics [40], and reduced lung function among children who spend more time outside [41]. More recently, research from the California Children's Health Study found that prolonged exposure to traffic pollution, including NO_2_, increases the incidence of childhood asthma [42,43]. Taken together, there is growing support for the relevance of traffic related pollution in the exacerbation and development of asthma.

The ambient pollutant that has been most frequently associated with asthma hospitalizations has been ozone. In our study, an association between ozone and asthma ED visits was observed in patients of all ages; this association was strongest among those 5 – 14 years of age, while not statistically significant in other age ranges. Ozone (O_3_) is formed from photochemical reactions between NO_X _and volatile organic compounds in the presence of sunlight. Ozone levels are highest on warm sunny and calm days, with exposures peaking in mid-afternoon. Controlled laboratory studies have shown that O_3 _can invoke acute lower inflammatory responses in both healthy and asthmatic subjects, however, asthmatics appear to experience more severe responses [44]. Given that the oxidant capacity of NO_2 _is smaller than that for O_3 _[45], our finding of a stronger association with NO_2 _and asthma visits is somewhat surprising. However, similar patterns have been observed in several recent asthma studies that have evaluated both pollutants [14,46]. Recently, an eight city panel study of 990 children found that NO_2 _and CO were strongly related to asthma exacerbations while no such association was noted for O_3 _[47]. Differences in meteorology, the complex mixture of pollution between regions, and the possibility of a threshold effect for ozone [48] may contribute to equivocal findings reported in different regions.

Emergency department studies of asthma have also evaluated the role of ambient particulate matter. Particulate air pollution is a mixture of solid particles and liquid droplets that can differ considerably in origin, size, and composition. Particulate matter includes aerosols, smoke, fumes, dust, ash and pollen. Fine particulate matter, which comprises those particles with an average aerodynamic diameter of 2.5 microns or less, has been studied more of late because it can better penetrate the respiratory system than particles of larger size. Positive associations between particulate matter and hospital visits for asthma have been reported in many international studies [20,46,49-56], but not all [9,19,34,47,57,58]. Future short-term health effect studies of ambient pollution need to better isolate the biologically important constituents, and physical properties of particles that invoke responses in persons with asthma.

The validity of our findings relies on the accuracy of diagnosing asthma within the ED, and this accuracy is known to vary by the patient's age. As mentioned before, we excluded asthma ED visits among children less than two years of age as it is often confused with bronchiolitis [59]. In older patients, while clinicians in theory are able to distinguish between asthma and chronic obstructive pulmonary disease (COPD), some diagnostic misclassification does occur [59,60]. To evaluate the extent that such misclassification affects our presented risk estimates, we evaluated the association between air pollution and COPD visits in the elderly. We found that outdoor levels of air pollution were unrelated to ED visits for COPD in our patient population. This indicates that two different disease entities are being captured through the diagnostic patterns in place in the Edmonton area hospitals. However, the misdiagnosis of COPD as asthma would serve to underestimate the strength of our associations.

We found that the associations were strongest between outdoor air pollution and ED visits for asthma among children between 2 – 4 years of age, and among the elderly (= 75). Children are widely regarded to be a susceptible population for air pollution health effects for several reasons. They have higher minute ventilation and higher levels of physical activity, spend more time outside than adults, and their peripheral airways more susceptible to inflammatory narrowing [61]. In addition, they retain a disproportionately higher amount of air pollution per unit body weight than adults [62]. Factors that may increase the susceptibility of the elderly to air pollution include: higher airways deposition rate of particulate matter, deficits of dietary factors such as antioxidants, and compromised immune systems due to comorbidities and increased medication use [13]. While further work is needed to evaluate how air pollution differentially affects the exacerbation of acute asthma by age, diverse findings from previously conducted Canadian studies highlights the continued need to look at both gaseous and particulate phase component of the air pollution mix.

The risk estimates presented here have been adjusted for meteorological effects of temperature, and relative humidity. They have also been adjusted for daily ED counts for influenza in order to control for viral respiratory seasonal epidemics. The case-crossover study design is also effective in controlling for the influence of individual-level risk factors that are unlikely to vary over short time intervals. For asthma, such factors are numerous and include: age, sex, cigarette smoking, household pets, and genetic predisposition to asthma. While cigarette smoke has been identified as an important risk factor for asthma, it is unlikely that it would confound our results as these exposures, as suggested by recent analyses of Canadian national survey data, are not related to with outdoor air pollution levels from fixed sited monitoring station [63]. Similarly, indoor sources of NO_2 _from cooking and heating are unlikely to be correlated with outdoor sources over the short time interval of the study. Perhaps most importantly, the time-stratified case-crossover approach has also been demonstrated as a suitable method to control for time trends in both air pollution exposures and outcomes [26].

The case-crossover approach relies on the assumption that the event of interest, here ED visits for asthma, define the case intervals while no such visit can occur during the matched control intervals. This assumption can be violated under the scenario of recurrent events. For example, individuals may present themselves to the ED for asthma multiple times, and therefore, the control periods associated with some individuals could be misclassified. With the time-stratified design, this would occur if an individual had an ED visit for asthma on the same day of the week more than once in a given month. Unlike many other hospital-based case-crossover studies of recurrent outcomes, patient identification data were available for most visits; this allowed us to evaluate the extent of this possible bias. In our dataset for which patient identification data were available, approximately 33% of these patients visited the ED more than once over the study period. However, there were very few instances (n = 411) where an individual who visited the ED for asthma had a subsequent visit for asthma on the same day of the week, within the same month in a given year. Neither the exclusion of these matched sets, nor the re-coding of the control intervals to properly reflect the fact that these were case intervals changed the risk estimates in any appreciable way.

Our risk estimates are reliant on the use of air pollution levels derived from fixed-site monitoring stations. Measurement error from fixed site monitoring stations can occur from the devices themselves, or from an inability to account for heterogeneous pollution levels that exist spatially within the region. The magnitude of these measurement errors vary between pollutants. For example, pollutants such as NO_2 _exhibit tremendous spatial variability and have been shown to be correlated to traffic measures [64]. In contrast, meteorological conditions strongly influence the efficiency of photochemical processes that lead to ozone formation [65,66], and for this reason ground-level ozone air pollution is largely characterized on a regional-scale basis, rather than on an intra urban scale. While individual-level exposure estimates are generally recognized to be superior for evaluating risk of environmental exposures, as pointed out by Schwartz the use of a daily mean exposure for an entire city is relevant [67]. Namely, the mean of personal exposures among residents in that city is likely more highly correlated with central monitoring station than individual exposures. Recent work into the measurement error associated with the use of fixed site monitoring stations suggest that most of the difference between personal and fixed site monitoring measurements of exposure are Berkson error, and therefore, do not bias the risk estimates. The work by Zeger suggests that the remaining measurement error follows the classical error model, and therefore, the overall net effect would be risk estimates that are biased towards the null [68]. As a result, the measurement error associated with the use of fixed site monitoring stations is not the source of the positive associations found in our study population.

Along the same lines, aeroallergen levels likely varied within the Edmonton census area. One sampling device was used to infer daily aeroallergen levels. We expect that the mixing of spores in air, and transport by wind provides a more uniform mixture of aeroallergens throughout the study region. Because of this, and the fact that people are not stationary, we feel that the use of one sampling device is a valid means to represent daily aeroallergen levels in the study region. Further support for this comes from a sampling study that found high correlations in pollen counts between two sampling sites located 5.6 km apart [69].

Exacerbations of asthma are often caused by viral illnesses, exposure to irritants or allergens. In this study, we partitioned ED visits into two seasons, April to September and October to March. Daily monitoring of aeroallergen indicate that their relevance pertains strictly to the period between April and September. In contrast, as evidenced by seasonal patterns in the number of daily visits for influenza, a viral etiology predominates the winter period. Like others, we modeled the daily number of visits for influenza to control for seasonal viral respiratory epidemics [20,70,71]. While the frequency of daily influenza visits were correlated with the number of asthma visits during the winter, the addition of this term produced no appreciable change in the air pollution risk estimates. Similarly, aeroallergen levels did not confound the air pollution risk estimates between April and September.

Air pollution levels were generally higher in the period between October to March, than April to September where associations with asthma ED visits were evident. Differences in the air pollution risk estimates between the two seasons themselves may possibly be explained in part by differential exposure misclassification. As Edmonton-area residents spend a greater proportion of their time outside during the spring and summer seasons, fixed-site monitoring data likely more accurately reflects the average exposure to ambient pollution in the summer. Therefore, if the association between air pollution and asthma is real, there would be greater attenuation in risk estimates for winter time exposures.

Finally, it is important to note that this study undertook a large number of comparisons as we explored associations between multiple ambient measures of air pollution pollutants using several types of metrics, across different age groups and seasons. Due to the large number of statistical tests performed, the chances of detecting a spurious finding are increased. We did not change our p-values to take into account the multiple testing performed in this study as such an approach has criticized for introducing more problems than they are intended to solve [72]. Despite the large number of tests performed, it is also important to recognize that our findings of stronger association in the young and elderly are consistent with the hypotheses we had a priori .[30,31].

## Conclusion

In conclusion, this study implicates ambient pollution, particularly NO_2_, as an important contributor to asthma morbidity between April and November, particularly in young children and elderly. This finding persisted after adjustment for meteorological variables, control for seasonal viral epidemics, as well as outdoor levels of aeroallergens. Efforts to mitigate these exposures should be considered, particularly in light of past initiatives that have produced tangible health benefits [30,31].

## Abbreviations

CH: Capital Health;

CI: Confidence interval;

CO: Carbon monoxide;

COPD: Chronic obstructive pulmonary disease;

ED: Emergency Department;

ICD-9: International Classification of Diseases, 9^th ^Revision;

IQR: Interquartile range;

NAPS: National Air Pollution Surveillance;

NO_2_: Nitrogen dioxide;

NOx: Nitrogen oxides;

OR: Odds ratio;

PM_10 _Fine particulate matter with an aerodynamic diameter of less than 10 μm;

PM_2.5_: Fine particulate matter with an aerodynamic diameter of less than 2.5 μm;

SO_2: _Sulfur dioxide;

WHO: World Health Organization.

## Competing interests

The author(s) declare that they have no competing interests.

## Authors' contributions

All authors have made substantive contributions to this study. PV conceived of the design, guided the analysis of the data, coordinated access of the data with the collaborators, and played the lead role in the writing and revising this manuscript. LC was the biostatistician who performed the analysis, and contributed in the writing of the manuscript. BR arranged for the acquisition of hospital data for the purpose of these analyses, and contributed to the drafting and revising of the manuscript. FC coordinated the collection and provision of the aeroallergen data used in these analyses and contributed in the writing of this manuscript. All authors have read and approved this version of the manuscript.
